# An open data infrastructure for the study of anthropogenic hazards linked to georesource exploitation

**DOI:** 10.1038/s41597-020-0429-3

**Published:** 2020-03-11

**Authors:** Beata Orlecka-Sikora, Stanisław Lasocki, Joanna Kocot, Tomasz Szepieniec, Jean Robert Grasso, Alexander Garcia-Aristizabal, Marc Schaming, Paweł Urban, Glenda Jones, Ian Stimpson, Savka Dineva, Piotr Sałek, Konstantinos Leptokaropoulos, Grzegorz Lizurek, Dorota Olszewska, Jean Schmittbuhl, Grzegorz Kwiatek, Aglaja Blanke, Gilberto Saccarotti, Karolina Chodzińska, Łukasz Rudziński, Izabela Dobrzycka, Grzegorz Mutke, Adam Barański, Aleksandra Pierzyna, Elena Kozlovskaya, Jouni Nevalainen, Jannes Kinscher, Jan Sileny, Mariusz Sterzel, Szymon Cielesta, Tomas Fischer

**Affiliations:** 1 Institute of Geophysics, Polish Academy of Sciences, Warsaw, Poland; 2 ACC Cyfronet, AGH, Krakow, Poland; 3 Isterre, Grenoble Observatory, Grenoble, France; 4 Istituto Nazionale di Geofisica e Vulcanologia, Sezione di Bologna, Via Donato Creti 12, 40128 Bologna, Italy; 5 Université de Strasbourg, CNRS, IPGS-UMR7516 Strasbourg, France; 6 Keele University, Keele, UK; 7 Luleå University of Technology, Luleå, Sweden; 8 Helmholtz Centre Potsdam, GFZ German Research Centre for Geosciences, Section 4.2: Geomechanics and Scientific Drilling, Telegrafenberg, 14473 Potsdam, Germany; 9 Istituto Nazionale di Geofisica e Vulcanologia, Sezione di Pisa. Via Cesare Battisti, 53 - 56125 Pisa, Italy; 10 Central Mining Institute, Katowice, Poland; 11 Polska Grupa Górnicza S.A., Katowice, Poland; 12 Oulu Mining School and Sodankylä Geophysical Observatory, University of Oulu, Oulu, Finland; 13 Institut national de l’environnement industriel et des risques, Nancy, France; 14 Institute of Geophysics, Academy of Sciences, CR, Prague, Czech Republic

**Keywords:** Seismology, Geophysics, Tectonics

## Abstract

Mining, water-reservoir impoundment, underground gas storage, geothermal energy exploitation and hydrocarbon extraction have the potential to cause rock deformation and earthquakes, which may be hazardous for people, infrastructure and the environment. Restricted access to data constitutes a barrier to assessing and mitigating the associated hazards. Thematic Core Service Anthropogenic Hazards (TCS AH) of the European Plate Observing System (EPOS) provides a novel e-research infrastructure. The core of this infrastructure, the IS-EPOS Platform (tcs.ah-epos.eu) connected to international data storage nodes offers open access to large grouped datasets (here termed episodes), comprising geoscientific and associated data from industrial activity along with a large set of embedded applications for their efficient data processing, analysis and visualization. The novel team-working features of the IS-EPOS Platform facilitate collaborative and interdisciplinary scientific research, public understanding of science, citizen science applications, knowledge dissemination, data-informed policy-making and the teaching of anthropogenic hazards related to georesource exploitation. TCS AH is one of 10 thematic core services forming EPOS, a solid earth science European Research Infrastructure Consortium (ERIC) (www.epos-ip.org).

## Introduction

The exploitation of georesources and underground storage of liquids and gases can pose environmental hazards as they can induce seismicity and cause deformation of the ground surface. They can create new fractures that change rock-mass permeability and may cause groundwater contamination and/or air pollution from the emission of fugitive gas and particulate matter^1–11^. Transient strong motions from induced earthquakes can cause infrastructure losses, human injuries and fatalities^2,12^. The socio-economic impacts of such anthropogenic hazards (AH) are significant. These risks can threaten or prohibit the development of the associated industries, including those instrumental for the transition to a low-carbon future such as geothermal energy and carbon dioxide sequestration. Vital technological activities may have to cease without mitigation of the accompanying hazards. This is a particularly sensitive topic in the densely populated areas of Europe where such technological activities often take place close to inhabited areas^13,14^.

These AH are poorly understood despite intensive world-wide research. Among the reasons for this are the complexities of geological and geophysical processes, the diversity and time-variability of the industrial processes responsible for generating these hazards, the complex link with natural hazards (e.g. in the distinction between triggered and induced seismicity) and the commonly non-public, proprietary nature of the data. However, AH research requires a holistic and interdisciplinary approach, as well as access to integrated and standardized data. Comprehensive science-industry collaborative efforts to monitor effectively, analyze and evaluate anthropogenic seismicity and the resulting hazards are underpinned by data sharing and can have tangible benefits for both industry and society. Access to ‘big’ and ‘open’ data from numerous case studies across different georesource sectors is needed to facilitate deeper scientific insight, enable retrospective research and to improve transparency. To facilitate this, a community of European scientists have created the Thematic Core Service Anthropogenic Hazards (TCS AH), utilizing the framework of the European Plate Observing System program (EPOS).

TCS AH is a new cooperative research undertaking developed by European representatives from science and industry, with a transnational governance framework covering implementation, best practice and sustainability strategies, outreach and dissemination. TCS AH provides the framework for global-scale investigation of AH related to georesource exploitation, achieved through open access data and applications in accordance with FAIR (Findable, Accessible, Interoperable, Reusable) data principles^15^.

**Fig. 1:**
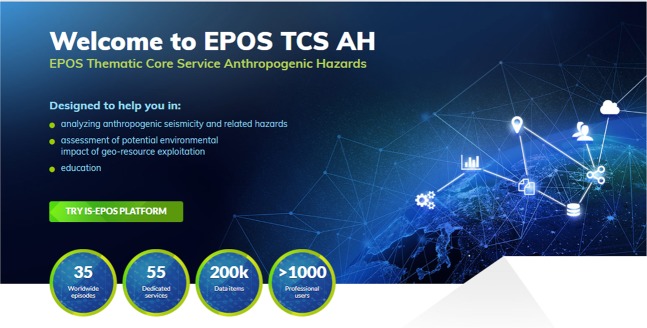
Landing web page of the EPOS TCS AH with access to IS-EPOS portal (tcs.ah-epos.eu).

TCS AH, one of the ten thematic core services of EPOS, has been developed within the framework of the European Commission’s infrastructural projects FP7-INFRASTRUCTURES-2010-1 (the EPOS preparatory phase, EPOS-PP), and H2020-INFRADEV-1-2015-1 (implementation phase, EPOS-IP) (Fig. 1). TCS AH coordinates the maintenance and development of the IS-EPOS Platform, initially established as a working prototype by the IS-EPOS POIG.02.03.00-14-090/13-00 project.

Data are gathered in thematic episodes. Each episode forms a comprehensive set of geophysical, industrial and environmental data related to induced seismicity originating from exploitation of a particular georesource. The platform is an innovative e-research environment for researchers, integrating the data and software applications, and providing a flexible virtual laboratory workspace for data processing, analysis and visualization. The platform supports collaborative functionalities, e.g. the sharing of user workspaces. The access to the integrated resources of the IS-EPOS Platform is open to all. Visitors to the IS-EPOS portal are able to preview the available resources without registration. Registered users, however, can utilize all the functionality of the platform.

## Methods

### System architecture

**Fig. 2:**
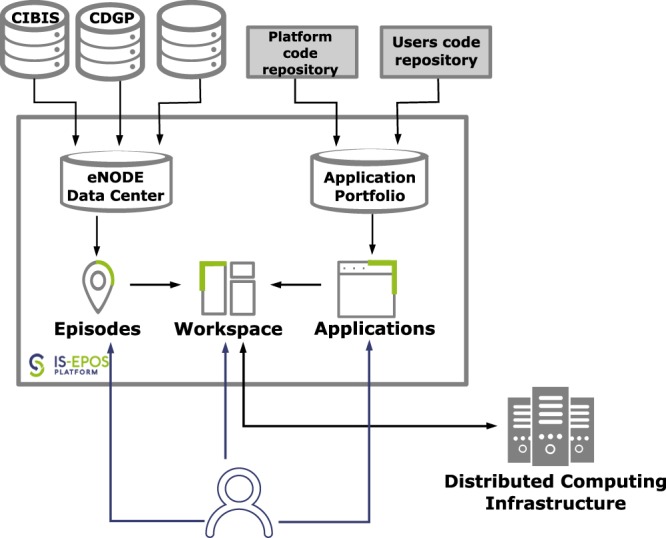
General architecture of IS-EPOS Platform with its main components: Episodes (data and metadata), Workspace and Applications. Episode data are stored in eNodes, fed into the eNode Data Center and then offered to the user. On their request, the data is loaded to a user’s Workspace. Application codes are stored in the code repositories, gathered in an Application Portfolio, and then shown to the user. These can then be loaded into a Workspace and executed on Distributed Computing Infrastructure.

The TCS AH research infrastructure is integrated and provided to the community through the IS-EPOS Platform. The platform (Fig. 2) provides access to:

- data and metadata gathered in the form of episodes with associated tools for data and metadata search and visualization;
- applications for online data processing;
- user-organized workspaces for storing and sharing data and applications.

In addition to the direct access via the IS-EPOS Platform, TCS AH will also be integrated with the EPOS-ERIC IT infrastructure. This will enable all EPOS users to access the data and functionality of the IS-EPOS Platform and to use them in multidisciplinary projects conducted with tools provided by the wider EPOS-ERIC IT Platform.

#### Data records and acquisition

Episode datasets related to investigations of particular anthropogenic hazard phenomena are maintained by participating institutions forming separate eNodes (e.g. CIBIS and CDGP eNodes). Data in eNodes are formatted in accordance with IS-EPOS Platform data-format specifications and described with associated metadata. The data are provided in unified formats, described in the IS-EPOS Platform documentation (docs.cyfronet.pl/display/ISDOC/Data + formats). The data formats are based, as far as possible, on existing standard formats (e.g. *seed, mseed, QuakeML, InventoryXML*), but for the types of data for which there are no established standard formats, the platform uses custom Matlab-based formats: catalog, GDF (*Generic Data Format*) and MDDF (*Multi-Dimensional Data Format*). The GDF and MDDF files have been designed as smart structures to store diverse scientific data, for example, water quality, air quality, industrial production data and geological/geophysical data. These data formats are very widely used and easy to use for further processing. GDF and MMDF files contain the identity of the geographic coordinate system in which data are stored, the time zone in which the time is determined, and information about the stored data, such as units, data type, and names of variables with descriptions. These metadata are stored in the CRF field (Coordinate Reference System) of the Matlab structure. Geographical data are usually stored using the WGS-84 ellipsoid reference coordinate system. Coordinate conversion to WGS-84 is a part of the IS-EPSOS Platform data integration process before the data undergoes thorough quality control and made available to users. There are also a number of format converter applications on the IS-EPOS Platform, which allow conversion from the internal formats to user-selected formats when the data is downloaded.

The associated metadata make the data discoverable and searchable, provide additional information about the content and origin of a specific data item and provide information on any access policy. Both data and metadata are subject to quality control (see: Technical Validation section below). Every episode published on the IS-EPOS Platform has a unique DOI for citation and direct access to the original data source, which is crucial under FAIR data principles. All the metadata coming from different eNodes are collected and indexed in the eNode Data Center.

#### Applications

**Fig. 3:**
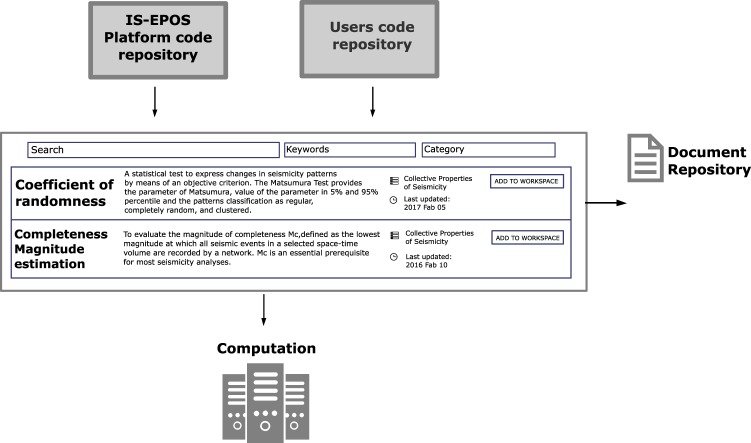
IS-EPOS Platform applications, combined from the official code repository, as well as from a custom user code repository. The applications have references to associated publications stored in the document repository.

Applications allow the processing of data from the IS-EPOS Platform as well as that uploaded by the user. Applications range from simple data management routines to advanced services for specialized data analysis. The latter are software packages developed, maintained and published by researchers. Future development of the IS-EPOS Platform should enable users to run their own data-analysis scripts within the platform (Fig. 3).

#### Workspace

**Fig. 4:**
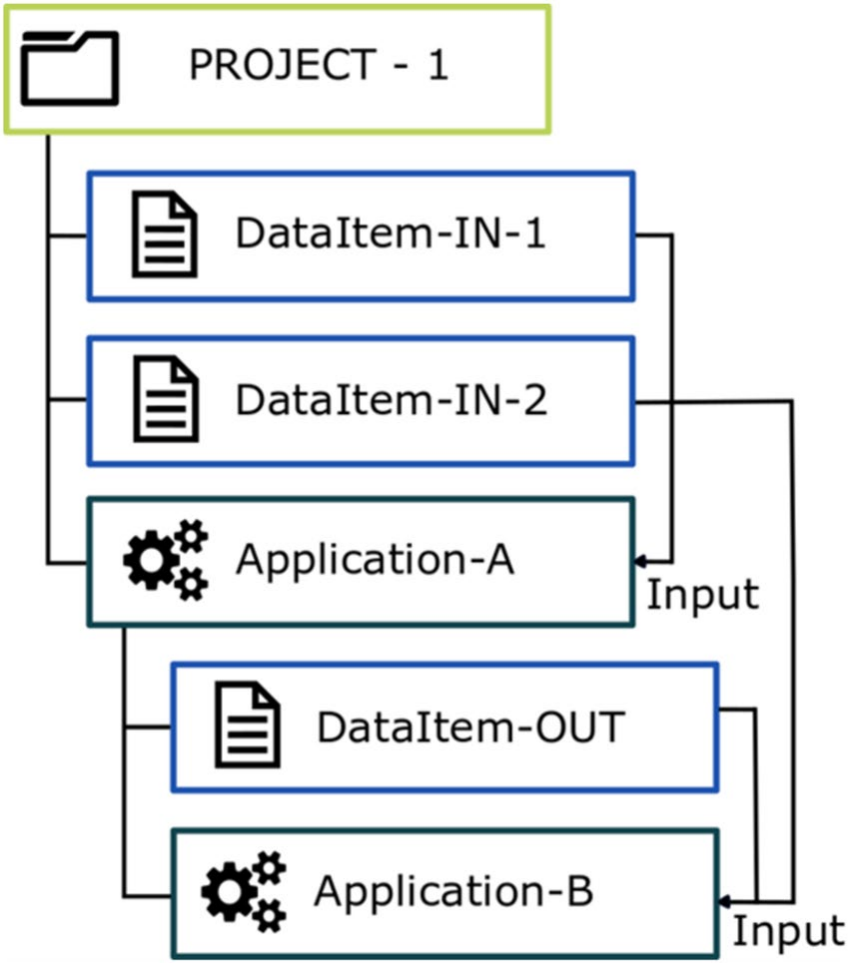
Schema of a sample organization of data and applications in workspace.

**Fig. 5:**
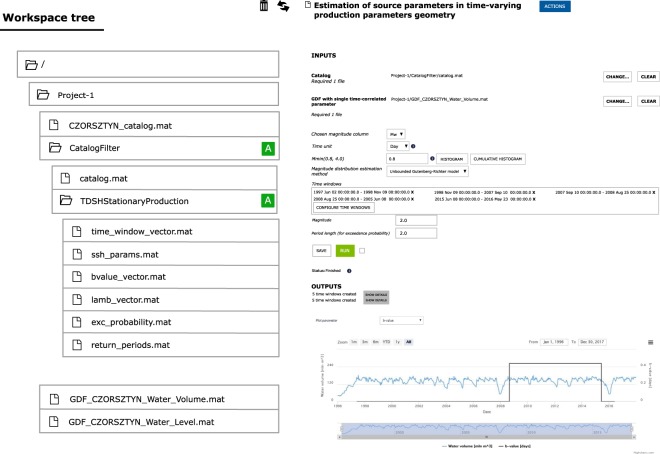
Sample application localized in a workspace, displaying the form of parameters, computation status and resulting visualization. The resulting data are stored in the application directory within a workspace tree, on the left.

The workspace provides the user with a framework to organize data and applications into integrated scientific projects. Data in workspaces are represented as files and they can be organized within a hierarchy of directories (Fig. 4). Each data file has its own metadata information allowing the user to match compatible applications with it. Users can add applications to their personal workspace in order to execute them on selected data and capture the resulting output. An application in a workspace is represented by a special kind of directory. This directory both organizes the data and provides an interface to handle data processing parameters, trigger the computation, as well as visualize, preserve and export the processed output (see Fig. 5). The processing itself is delegated to a distributed computing infrastructure (cloud or high-performance computers). The output of an application execution is stored in the application directory and may become input for another application. Consequently, a series of applications with their resultant data may be combined to form a workflow.

All data stored in the user’s workspace can be shared with other users of the platform, either in an editable or read-only mode. Similarly, the application settings and processing workflows can be shared.

#### Integration with EPOS-ERIC IT

**Fig. 6:**
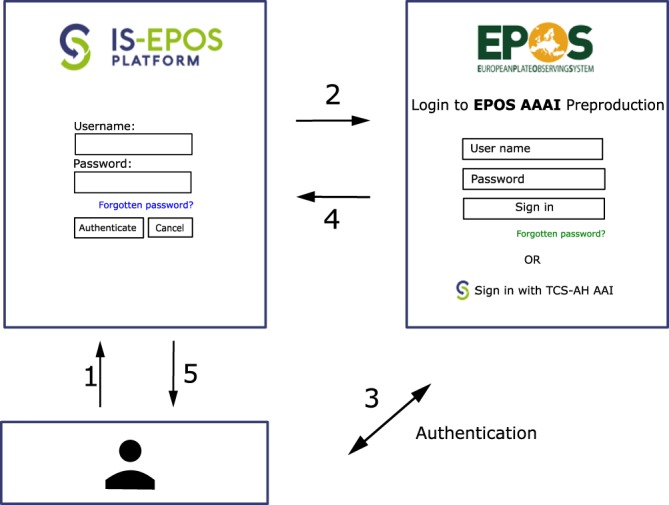
The process of user authentication to the IS-EPOS portal using an EPOS AAAI account. Numbers refer to the flow of actions.

Ultimately, all ten thematic core services of EPOS, including TCS AH, will be integrated into the EPOS-ERIC IT infrastructure known as Integrated Core Services, ICS, where users will be able to discover, access and process multidisciplinary datasets available from the individual TCSs. TCS AH is integrated with the EPOS Authentication, Authorization and Accounting Infrastructure (EPOS AAAI). This allows TCS AH users to log on to the ICS platform with their existing credentials and wider EPOS users to access TCS AH data and applications. There is also an authorization mechanism to recognize various user roles and attributes through the EPOS AAAI in order to grant access to specific resources within the platform. This is implemented using standard protocols such as OpenId Connect and OAuth 2.0. The interactions involved in these protocols are depicted in Fig. 6.

Another aspect of integration with the ICS is related to the metadata that describes data maintained by the individual TCSs. These metadata are harvested and converted by the ICS to make the data discoverable and usable in a uniform way across the different EPOS TCS platforms.

### User engagement and science-industry partnership

The AH community is comprised of stakeholders from academia, industry, educational institutions, NGOs, the public and government organizations. Spreading awareness of AH phenomena and the services offered by TCS AH has involved building an online presence (project website content, social media communications, online newsletters), face-to-face interaction (outreach events, scientific workshops, IS-EPOS Platform training workshops) and scientific dissemination (peer reviewed publications, conference presentations and participation at events in, and related to, the field of anthropogenic hazards). Approximately 10 training workshops have taken place with over 200 AH community stakeholders from the scientific community being trained to use the IS-EPOS Platform across 7 countries during the course of the EPOS-IP project. In addition to gaining feedback on the IS-EPOS Platform, the training events organized by TCS AH and important face-to-face stakeholder engagements have fostered the development of new ideas for the use of the integrated research infrastructure. The IS-EPOS Platform has also been used as a teaching tool in an educational project aimed at junior high and high schools called ERIS – Exploitation of Research Results in School practice, funded with support from the European Commission within the ERASMUS + Programme. It has also been integrated into university teaching as an educational and effective research resource at several of the TCS AH consortium institutions.

By August 2019, the IS-EPOS Platform had more than 1000 registered users (an increase of 720% since the launch of the EPOS-IP project in 2015). To stimulate further the use of the platform and the provision of services, the TCS AH consortium is open to new collaborations. Data and/or software from new potential partners, from on-going or past projects are welcome. Collaboration is also sought with companies producing equipment for seismic activity monitoring or accompanying processes. The ambition is not only to be a provider of an e-research environment but also to perform intercommunity social functions like project brokering and developing opportunities for new interdisciplinary and international collaborations.

### Governance and future perspectives

The TCS AH community is organized around a TCS AH consortium of 13 partnering institutions (suppliers of data and/or services) from 8 European countries. The work of the consortium is governed by a Consortium Board consisting of representatives of all the partners and with a director elected for 5 years by a majority of the board. Five sections have been established within TCS AH: (a) Implementation of services; (b) Administration, law & accounting; (c) Episodes integration and application implementation; (d) Promotion and dissemination; (e) Projects & partnership. There are two external committees, the Data Provider and User committees which are composed of the main representatives of the respective communities, selected by the majority of the board to advise the director. An Innovation Advisory Committee consisting of stakeholders from academia, industry, local and central administration bodies, society and others, invited by the Consortium Board, consults on the decision-making processes behind TCS AH developments. Membership of the TCS AH consortium is open to new partners.

The plan is to establish TCS AH as a coherent coordinating framework for the AH community, together with a robust research infrastructure to develop strategic research capacity for addressing AH challenges. The goals are to facilitate new discoveries, connect the stakeholder community, boost public understanding of AH, develop outreach materials for the public and future scientists, stimulate innovation through knowledge transfer, provide solutions to industrial partners and engage in a three-way transfer of knowledge between industry, science and society.

## Resource Description

### Data records and acquisition – cross-national episode eNodes

#### IG PAS eNode – CIBIS

**Fig. 7:**
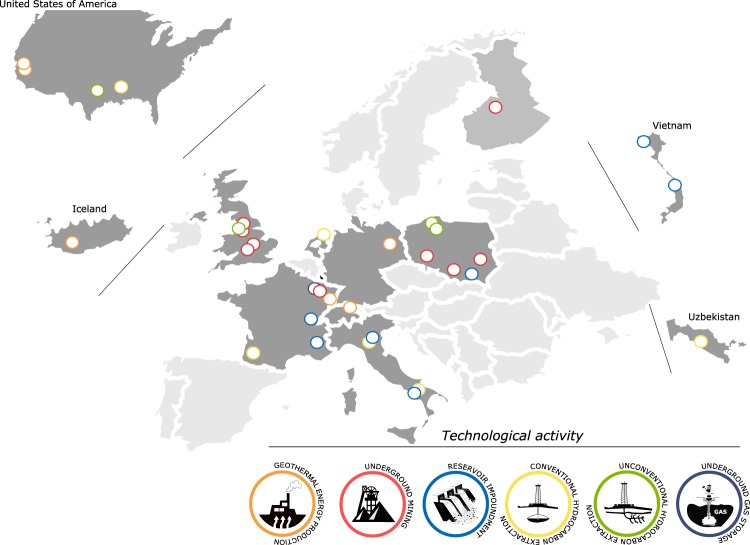
Geographical distribution of episodes stored in CIBIS and CDGP.

The IG PAS eNode ‘Induced Seismicity Research Infrastructure Center’ (CIBIS) is located in Krakow, Poland and managed by the Institute of Geophysics Polish Academy of Sciences, where more than 30 AH episodes from various projects are stored and maintained (Figs. 2 and 7), covering a broad variety of industrial activities. The majority of the episodes relate to induced seismicity and ground instability from underground mining (10 episodes). Water reservoir impoundment is the second most represented industrial activity (6 episodes). Next are conventional hydrocarbon extraction, underground fluid storage and geothermal energy production episodes (5 data sets, including one complex episode involving CO_2_ storage). Episodes on unconventional hydrocarbon extraction related to fracking (4 data sets) are also stored in CIBIS with data included from the H2020 projects SHEER (Shale gas Exploration and Exploitation Induced Risks) and S4CE (Science 4 Clean Energy). The integrated episodes are located mainly in Europe (24), but also in Northern America (3) and Asia (3) (Fig. 7).

**Fig. 8:**
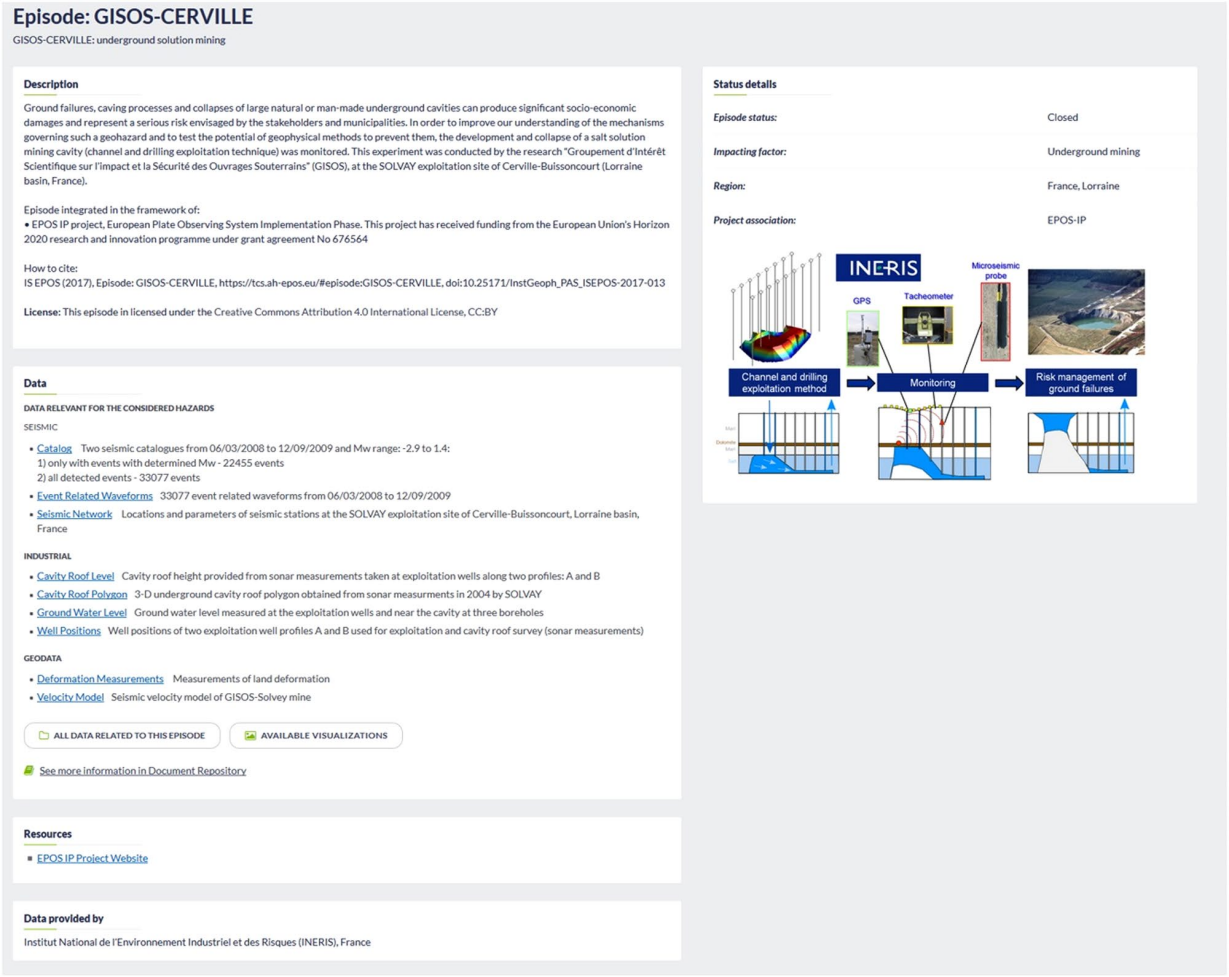
Example of data organization within an episode on IS-EPOS Platform.

Data within the episodes mostly include geophysical observations related to the anthropogenic hazard acquired directly from instrumental measurements as well as industrial data describing the processes potentially causing the hazard. Geophysical data include seismological event catalogs, ground motion catalogs and waveform data, but also water and air quality data from *in situ* and laboratory measurements. Geological data, including bedrock geology and tectonic features are also included, as well as geospatial and geotemporal data related to the causal activity. Every episode also contains a brief description of the locality and potentially hazard-inducing processes together with references and complementary documentation. A typical episode summary page is shown in Fig. 8.

#### CDGP eNode

The ‘Data Centre for Deep Geothermal Energy’ (Centre de Données de Géothermie Profonde - CDGP) archives data collected at the Upper Rhine Graben geothermal sites and distributes them to the scientific community, taking relevant intellectual property rights into account. It is located at the École et Observatoire des Sciences de la Terre (EOST, Université de Strasbourg, CNRS), and was created by the LabEx G-eau-thermie Profonde, a research program on deep geothermal energy founded by the French Ministry of Research and Education under the “Laboratories of Excellence” initiative. It operates as a standalone data centre (cdgp.u-strasbg.fr) but has also been incorporated as an eNode of TCS AH, available through the IS-EPOS Platform.

Data distributed by the CDGP consist of seismological (catalogs, waveforms) and hydraulic data that were acquired during stimulation or circulation phases at the Soultz-sous-Forêts pilot plant during the research period (1988–2010). Other geophysical data (gravimetric, magnetic, InSAR, geodesy, velocity model, etc.) will be also added to the data store in the future, as well as other data from different projects (Rittershoffen, Illkirch, Vendenheim). Agreements with industrial partners allow the CDGP to distribute particular data to the academic community. The CDGP infrastructure directly shares as much information as possible such as episode metadata with the IS-EPOS Platform to avoid work duplication, however, some special tasks are necessary to complete the metadata information needed by the IS-EPOS Platform as well as the reformatting of certain data.

### Applications

The IS-EPOS Platform provides users with a range of data analysis and manipulation routines. These are mostly (but not exclusively) Matlab or Python programs that allow analysis, processing and visualisation of data available on the platform or imported into it^16,17^. Currently, 44 applications are integrated into the IS-EPOS Platform grouped into 13 thematic categories (Table 1) in order to facilitate particular research analyses related to anthropogenic hazards. Some programs can be freely downloaded under the terms of a GNU General Public License.

**Table 1:** Applications available on the IS-EPOS Platform.

Collective Properties of Seismicity	Anderson-Darling test for exponentiality of inter-event time
	Coefficient of randomness
	Completeness Magnitude estimation
	Magnitude conversion
	Priestley-Subba Rao (PSR) test
Converters	CSV to Catalog converter
	Catalog to ASCII converter
	Catalog to Vectors converter
	GDF to Vectors converter
	GDF to XLS converter
	Catalog to XLS converter
	Ground Motion Parameters Catalog builder
	Time Series builder
	Seed converter
Correlation Analysis	Autocorrelation
	Cross-correlation
Data Processing Applications	Basic Vector Operations
Download Tools	Signal download tool
	Waveform download tool
Earthquake Interactions	Earthquake interactions: Georesource scale
	Earthquake interactions: Mainshock scale
	Earthquake swarm (reshuffling analysis)
	Time correlated earthquakes (Seasonal trends)
Event Detection Algorithms	Template-matching based detection algorithm
Filtering Tools	Catalog filter
	Estimation of source parameters in time-varying production parameters geometry
	Ground Motion Prediction Equations
	MERGER: Dynamic risk analysis using a bow-tie approach
Probabilistic Seismic Hazard Analysis	Source size distribution functions/Stationary Hazard
	Stationary Hazard: Exceedance Probability
	Stationary Hazard: Maximum Credible Magnitude
	Stationary Hazard: Mean Return Period
	Time dependent hazard in mining front surroundings
	Time dependent hazard in selected area
Seismogram Analysis Tools	Seismogram picking tool
Source Parameter Estimation	Effective stress drop estimate
	Estimation of source parameters in time-varying production parameters geometry
	FOCI
	Localization
	Mechanism: Full Moment Tensor
	Mechanism: Shear Slip
	Spectral Analysis
	Waveform-based seismic event location
Stress Field Modelling	Stress inversion
Visualizations	Estimate of maximum possible magnitude for reservoir triggered seismicity
	Fracture Network Models - Mechanical Stresses
	Front Advance histograms
	Integrated Google Maps data visualization
	Seismic Activity with Front Advance

The integrated applications comprise software tools which implement peer-reviewed techniques designed for dealing with specific scientific issues. These tools enable the user to perform research operations and analyses based on either seismic catalog data (e.g. correlation analysis, probabilistic seismic hazard assessment, earthquake interactions, stress field modelling and collective properties of seismicity) or waveform recordings (e.g. seismic event detection algorithms, event location, source parameter estimation including moment tensor inversion, and spectral analysis). In order to achieve more flexible workflows and a user-friendly environment, these applications are supported by a variety of data handling and visualization applications for performing basic parameter operations and data filtering, converting catalog and waveform data formats and graphically visualizing seismic as well as operational data. The results of the applications can be downloaded, reformatted, visualized, and used as input for further analysis. The user can create individual projects within their personal workspace, which can then be shared with other colleagues or research teams.

New applications are being developed and added in the Continuous Integration and Continuous Delivery (CI/CD) scheme bringing additional scientific potential to the platform, improving the user experience and increasing the ability to conduct advanced studies in the field of anthropogenic hazards. Advanced applications (template matching and multi-scale array-based algorithms for microseismicity detection and location, statistical toolboxes for magnitude complexity analysis and seismic event clustering) are being developed and are ultimately planned to be integrated into the IS-EPOS Platform as part of the project SERA —H2020-INFRAIA-2016-1.

In order to make the IS-EPOS Platform more inclusive and user friendly, future development plans for the platform include the introduction of the choice of user language. The ultimate goal is to provide the users with the ability to upload their own code scripts and to modify the source code of existing applications according to their specific requirements.

### Document repository

The IS-EPOS Platform provides a repository of documents associated with episodes and applications. This e-repository is powered by EPrints 3 (GLU license, www.eprints.org) which has been developed by the School of Electronics and Computer Science at the University of Southampton, UK. Thus the IS-EPOS Platform documents support OAI 2.0 with a base URL of tcs.ah-epos.eu/eprints/cgi/oai2. The front page is organized so that legal requirements, the user guide, guidance on how to cite the IS-EPOS Platform and other references to episodes and applications, can be directly accessed. The platform legal regulations contain documents regarding data policy, the data management plan and TCS AH governance services.

The user guide section consists of technical documents with instructions to assist using the IS-EPOS Platform, with a step-by-step tutorial on how to access and make use of particular applications. Links to the user guide for specific applications are also available from the application level. The user guide also includes guidance about data formats and the vocabulary of the IS-EPOS Platform.

The IS-EPOS Platform documents are thematically organized and classified, consisting of links to relevant articles, books, book sections, conference and workshop items or reports. There are currently 858 items in the e-repository. The internal search engine allows browsing using filters and search criteria. Items are classified according to episode, type of hazard-inducing phenomenon, monitoring technology, and geographic region. Consequently, the elements of the document repository can be readily accessed by applying specific filters. In addition, references relating to particular episodes and applications are also available as a link from their respective sections on the platform. There is also a collection of links to documents that reference the IS-EPOS Platform itself. (tcs.ah-epos.eu/eprints/id/saved_search/5).

### Integration of new episodes data – quality control procedure

**Fig. 9:**
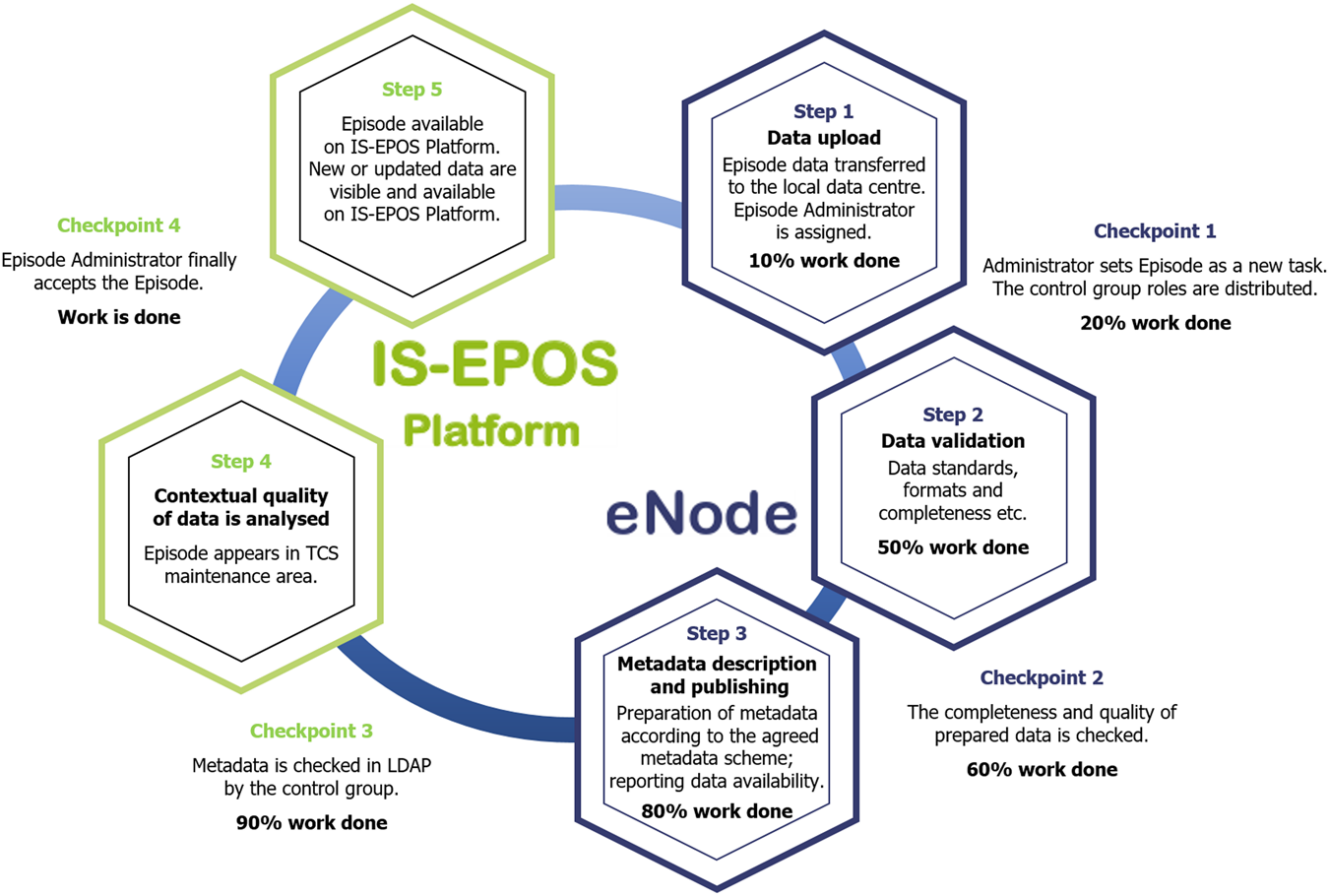
Quality Control Workflow of the AH Episode Access Service.

Quality control of the integrated and published episode data is carried out according to the diagram shown in Fig. 9 which illustrates the data processing steps and relevant control points. An open-source project web system is used to manage the episode integration process (Redmine, www.redmine.org). This allows the assignment and management of personnel responsible for the episode integration, as well as tracking the progress of that integration. The uploaded data are prepared, checked and validated to ensure that they conform to commonly used standards and formats. Any inconsistencies in the data structure are identified by the quality control team. Once the data and metadata are approved, they are made available on the IS-EPOS Platform test portal from the external source. The episode quality and integrity is then reviewed together with the data owner before the data and associated metadata is made public on the IS-EPOS Platform.

## Application Validation

Any code intended to be integrated as an IS-EPOS Platform application has to pass a two-step validation before it is released in the application portfolio. First, before the integration process starts, a technical validation aims to determine whether the code meets the technical requirements of integration. The program is checked to ascertain if it can be run on distributed computing infrastructure, if the licences (of the code itself or the libraries it uses) allow for its use on the IS-EPOS Platform and if the input and output file formats are compatible with the platform. The second step of validation includes thorough testing of the integrated application before releasing it to the public. This is an evaluation of the interface and the application potential by testing the application with various data samples available on the platform, an assessment of the application potential to build chains with other integrated applications to form a workflow, an evaluation of the usefulness of the application documentation, and the identification of bugs. This stage of validation may be an iterative process, including improvements to the code and reassessment of its functionality.

## Usage Examples

### Example of application available on IS-EPOS platform

Complementary to the data-handling, processing and visualization tools, the IS-EPOS Platform also provides a set of tools for tackling complex multi-hazard risk (MHR) scenarios usually found in activities related to the development of georesources. A service called “Simulator for Multi-hazard risk assessment in ExploRation/exploitation of GEoResources” (MERGER) has been designed to provide tools for probabilistic multi-hazard analyses^18^.

The main objective of this tool is to provide a quantitative model for performing highly specialized MHR assessments in which risk pathway scenarios are structured using a bow-tie approach, which implements the integrated analysis of fault trees and event trees. The methodology implemented in this service is suitable for performing dynamic environmental risk assessments. This is characterized by the bow-tie structure coupled with a wide range of probabilistic models flexible enough to consider different typologies of phenomena, the Bayesian implementation for data assimilation, the handling and propagation of modelling uncertainties and the possibility of integrating data derived from integrated assessment modelling.

**Fig. 10:**
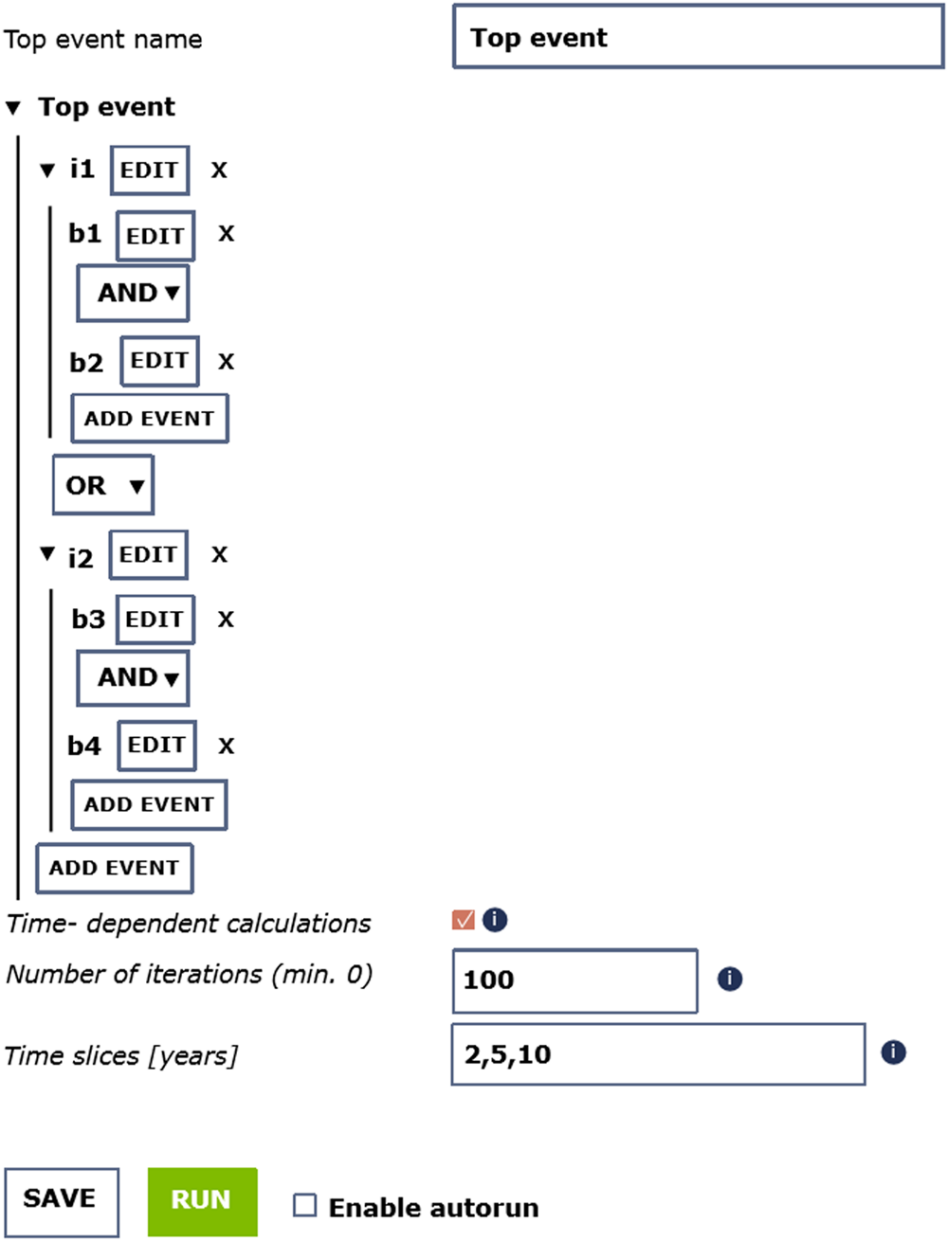
View of the graphical user interface available for the input of a fault tree (data and logic structure) in MERGER.

Once MHR scenarios have been identified and structured according to a bow-tie logic, both the scenario structure and the data can be loaded through a user-friendly graphical interface which guides the user through this process (Fig. 10).

**Fig. 11:**
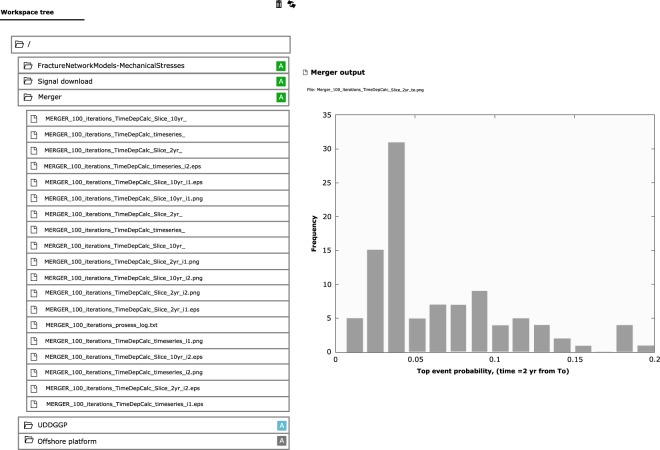
Example of the output produced by MERGER and shown on the IS-EPOS Platform for the top event of a fault tree. The selected output is displayed directly in the workspace, other results can be selected in the workspace tree on the left.

The output produced by this tool provides probabilistic assessments of the fault trees and event trees that were implemented for analyzing a given MHR scenario. Data output is provided numerically (in a log file) and graphically (e.g., through histograms) for each event of interest (as for example shown in Fig. 11).

### Use case 1: Studying correlation between injection rate and seismicity rate

**Fig. 12:**
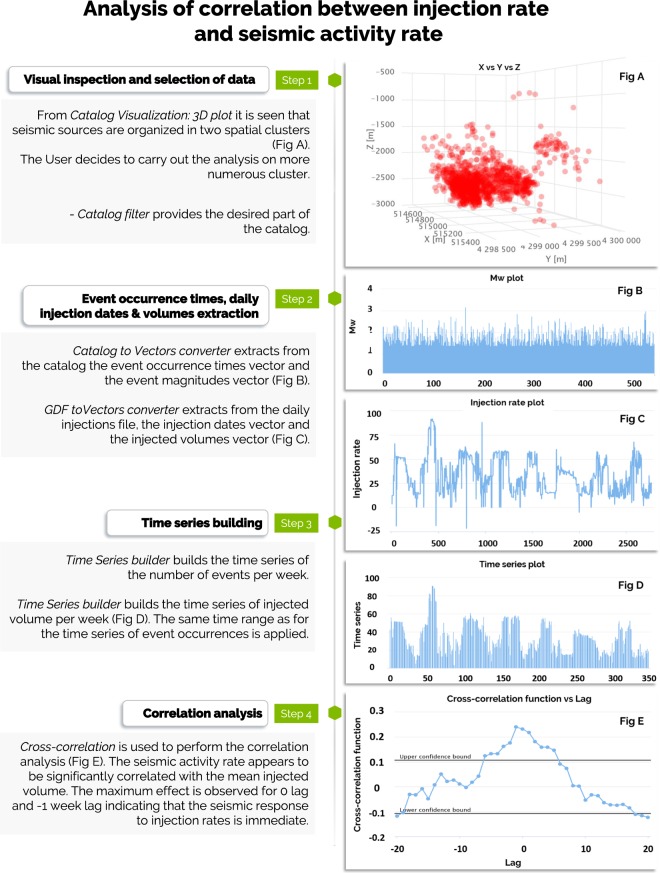
Workflow of analysis of correlation between injection rate and seismic activity rate during geothermal energy production.

Discovering the nature of the relationships between induced seismicity and the industrial factors that are its cause is probably the most important goal of anthropogenic seismicity studies because only the understanding of such relationships can lead to the development of methods to mitigate the anthropogenic seismic hazard. The example in Fig. 12 presents the steps in investigating if and how the seismicity induced by geothermal energy production correlates with the rate of fluid injection. The data for this use case has been taken from the episode “The Geysers Prati 9 and Prati 29 cluster”^19^ on the IS-EPOS Platform (tcs.ah-epos.eu/#episode:THE_GEYSERS_Prati_9_and_Prati_29_cluster, doi:10.25171/InstGeoph_PAS_ISEPOS-2017-011) and the analysis makes use of applications integrated on the platform.

### Use case 2: Integrated visualization of artificial lake water level changes and triggered seismicity

**Fig. 13:**
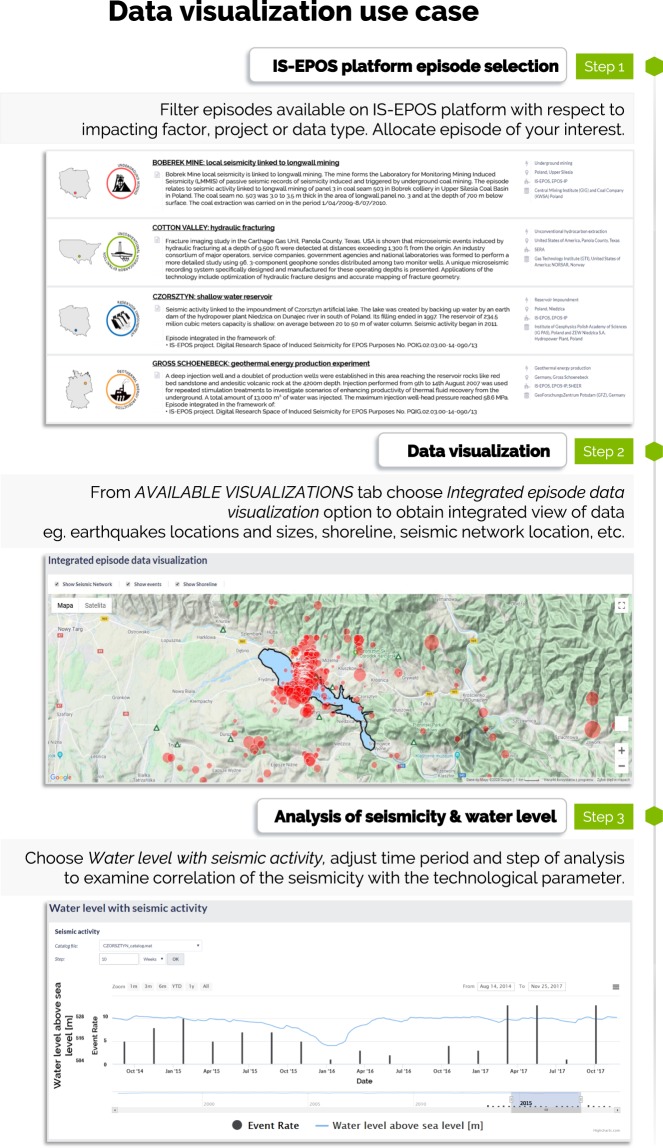
Example of the integrated visualization of water reservoir triggered seismicity and the triggering technological operations.

Comprehensive visualization tools available on the IS-EPOS Platform enable integrated visual inspection of multidisciplinary data. The example presented in Fig. 13 is taken from the episode “Czorsztyn” of seismicity triggered by the impoundment of an artificial lake https://tcs.ah-epos.eu/#episode:CZORSZTYN, doi:10.25171/InstGeoph_PAS_ISEPOS-2017-004. It shows the steps leading to the visualization of the triggered earthquake locations and magnitudes, the location of seismic recording stations, the lake shoreline on a topographic map background, and the occurrence times and sizes of the earthquakes superimposed on the lake water level time series. The IS-EPOS Platform maps data in WGS-84 ellipsoid coordinates, so datasets from various regions of the globe can be directly compared. Detailed information about the coordinate system used for a particular dataset is included in a file structure description and in the metadata.

### Use case 3: Seismic hazard assessment

**Fig. 14:**
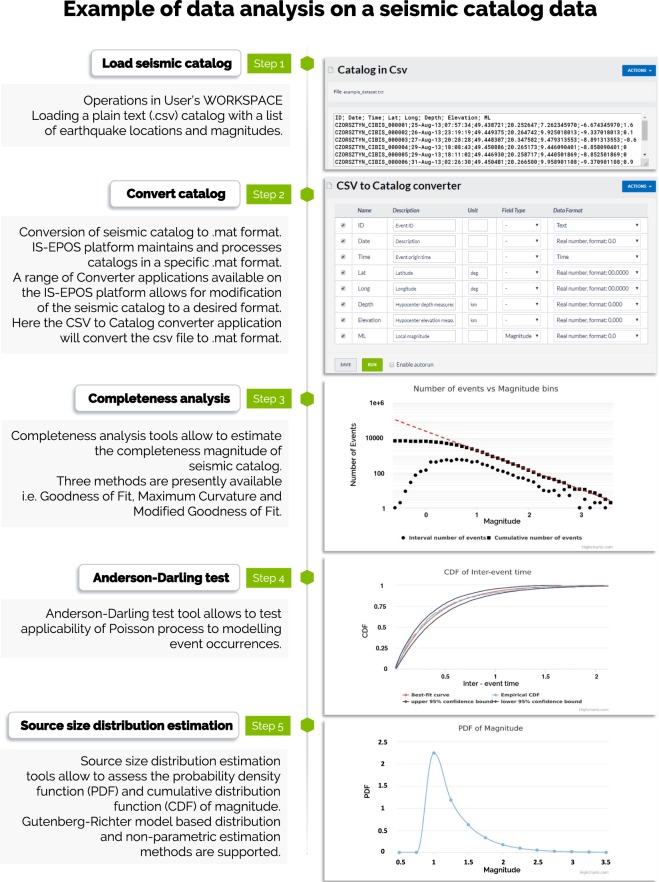
Illustration of the workflow for seismic hazard analysis. Left - the workflow, right – platform screen snapshots.

The IS-EPOS Platform features enable complex scientific analyses. The workflow diagram in Fig. 14 shows steps of an example analysis towards seismic hazard assessment. The presented analysis starts with loading a seismic catalog in csv format into the user’s workspace. After converting the catalog into the internal IS-EPOS (.mat) format, the user carries out the analysis using various applications available on the platform. The user can execute advanced statistical applications that support both parametric and nonparametric methods, and perform analyses leading to the probabilistic seismic hazard assessment in a stationary or time-dependent mode. Applications implemented on the platform also allow performing a range of alternative analyses based on adopted scenarios. The user can conduct ground motion prediction, source parameters estimation, etc.

## Data Availability

The datasets of episodes are available on the IS-EPOS Platform of Thematic Core Service Anthropogenic Hazards: tcs.ah-epos.eu. In accordance with the EPOS Data Policy which is available at www.epos-ip.org; and in accordance with TCS AH Data Policy, which is available at www.tcs.ah-epos.eu; datasets and applications are licensed under the Creative Commons Attribution 4.0 International License, CC:BY.

## Code availability

Applications are available on the IS-EPOS Platform of Thematic Core Service Anthropogenic Hazards: tcs.ah-epos.eu. The source code of the platform itself is available at bit.ly/ISEPOSsourcecode.
