# Author Correction: An open data infrastructure for the study of anthropogenic hazards linked to georesource exploitation

**DOI:** 10.1038/s41597-020-0457-z

**Published:** 2020-04-07

**Authors:** Beata Orlecka-Sikora, Stanisław Lasocki, Joanna Kocot, Tomasz Szepieniec, Jean Robert Grasso, Alexander Garcia-Aristizabal, Marc Schaming, Paweł Urban, Glenda Jones, Ian Stimpson, Savka Dineva, Piotr Sałek, Konstantinos Leptokaropoulos, Grzegorz Lizurek, Dorota Olszewska, Jean Schmittbuhl, Grzegorz Kwiatek, Aglaja Blanke, Gilberto Saccorotti, Karolina Chodzińska, Łukasz Rudziński, Izabela Dobrzycka, Grzegorz Mutke, Adam Barański, Aleksandra Pierzyna, Elena Kozlovskaya, Jouni Nevalainen, Jannes Kinscher, Jan Sileny, Mariusz Sterzel, Szymon Cielesta, Tomas Fischer

**Affiliations:** 1 Institute of Geophysics, Polish Academy of Sciences, Warsaw, Poland; 2 ACCCyfronet, AGH, Krakow, Poland; 3 Isterre, Grenoble Observatory, Grenoble, France; 4 Istituto Nazionale di Geofsica e Vulcanologia, Sezione di Bologna, Via Donato Creti 12, 40128 Bologna, Italy; 5 Université de Strasbourg, CNRS, IPGS-UMR7516 Strasbourg, France; 6 Keele University, Keele, UK; 7 Luleå University of Technology, Luleå, Sweden; 8 Helmholtz Centre Potsdam, GFZ German Research Centre for Geosciences, Section 4.2: Geomechanics and Scientifc Drilling, Telegrafenberg, 14473 Potsdam, Germany; 9 Istituto Nazionale di Geofsica e Vulcanologia, Sezione di Pisa. Via Cesare Battisti, 53 - 56125 Pisa, Italy; 10 Central Mining Institute, Katowice, Poland; 11 Polska Grupa Górnicza S.A, Katowice, Poland; 12 Oulu Mining School and Sodankylä Geophysical Observatory, University of Oulu, Oulu, Finland; 13 Institut national de l’environnement industriel et des risques, Nancy, France; 14 Institute of Geophysics, Academy of Sciences, CR, Prague, Czech Republic

Correction to: *Scientific Data* 10.1038/s41597-020-0429-3, published online 11 March 2020

Following publication of this Data Descriptor it was found that co-author Gilberto Saccorotti’s surname was spelled incorrectly. This has now been corrected in both the HTML and PDF versions.

